# Prognostic and predictive values of CDK1 and MAD2L1 in lung adenocarcinoma

**DOI:** 10.18632/oncotarget.13252

**Published:** 2016-11-09

**Authors:** Yuan-Xiang Shi, Tao Zhu, Ting Zou, Wei Zhuo, Yi-Xin Chen, Ma-Sha Huang, Wei Zheng, Chen-Jing Wang, Xi Li, Xiao-Yuan Mao, Wei Zhang, Hong-Hao Zhou, Ji-Ye Yin, Zhao-Qian Liu

**Affiliations:** ^1^ Department of Clinical Pharmacology, Xiangya Hospital, Central South University, Changsha 410008, P. R. China; Institute of Clinical Pharmacology, Central South University, Hunan Key Laboratory of Pharmacogenetics, Changsha 410078, P. R. China; ^2^ Hunan Province Cooperation Innovation Center for Molecular Target New Drug Study, Hengyang 421001, P.R.China

**Keywords:** prognosis, biomarker, lung cancer, CDK1, MAD2L1

## Abstract

Lung cancer remains as the leading cause of cancer-related death worldwide, and lung adenocarcinoma (LUAD) is the most common histological subtype. This study aims to investigate biomarkers associated with cancer progression and prognosis of LUAD. We integrated expression profiles of 668 lung cancer patients in five datasets from the Gene Expression Omnibus (GEO) and identified a panel of differentially expressed genes (DEGs). Function enrichment analysis highlighted that these genes were closely associated with the carcinogenesis of LUAD, such as cell cycle, ECM-receptor interaction and p53 signaling pathway. Cyclin-dependent kinase 1 (CDK1) and MAD2 mitotic arrest deficient-like 1 (MAD2L1), two critical mitotic checkpoint genes, were selected for further study. Elevated expression of CDK1 and MAD2L1 was validated in an independent LUAD cohort. Kaplan-Meier analysis revealed that CDK1 and MAD2L1 expression was negatively correlated with both overall survival (OS) and relapse-free survival (RFS). In conclusion, CDK1 and MAD2L1 were adverse prognostic biomarkers for LUAD whose increased expression could render patients with LUAD a high risk of cancer recurrence and poor survival, suggesting that they might be applied as potential targets for LUAD treatment.

## INTRODUCTION

Lung cancer is the leading cause of cancer-related death worldwide, of which non-small cell lung cancer (NSCLC) accounts for approximately 85% cases [1]. NSCLC comprises three major histological subtypes: squamous cell carcinoma (LUSC), adenocarcinoma (LUAD) and large cell carcinoma. The most common type of NSCLC is adenocarcinoma, which comprises around 40% of all lung cancer [2]. Despite of advances in the diagnosis and treatment for NSCLC, the 5-year survival rate for advanced NSCLC remains poor. It is still urgent to identify sensitive and specific biomarkers that could predict tumor recurrence and prognosis to guide the treatment of NSCLC.

Genome-wide expression profiles have recently been used to identify prognostic signatures in patients with cancer [3–6]. However, some genes identified with prognostic implications in one cohort might be difficult to be verified in other cohorts [7, 8]. An explanation might be that the effects of genes with broad confidence intervals are difficult to confirm using a validation strategy, that is, when genes are identified as significant in one study, they are further tested for significance in separate subsequent studies with smaller sample sizes [9]. To address these issues, validation of the signature genes in several independent studies or distinct patient populations is necessary.

In the current study, we compared gene expression changes between tumor tissues and adjacent non-tumor lung tissues (NTL) using five datasets, and overlapped the differentially expressed genes, which could be specifically involved in development of LUAD. We identified 125 differentially expressed genes in LUAD that were common among all five profiles. In addition, we validated the overlapping genes in independent patient cohorts. Further, our function enrichment analysis showed that genes related with cell cycle were the most significantly enriched. Thus, in this study, we focused on CDK1 and MAD2L1, two critical mitotic checkpoint genes which play an important role in the mitotic process. We found that CDK1 and MAD2L1 were up-regulated in LUAD and directly correlated with the clinical pathological features. We further investigated and explored the prognostic value of CDK1 and MAD2L1 in LUAD. Our data indicated that high CDK1 or MAD2L1 expression was associated with poor prognosis of LUAD.

## RESULTS

### Identification of differentially expressed genes

**Figure 1:**
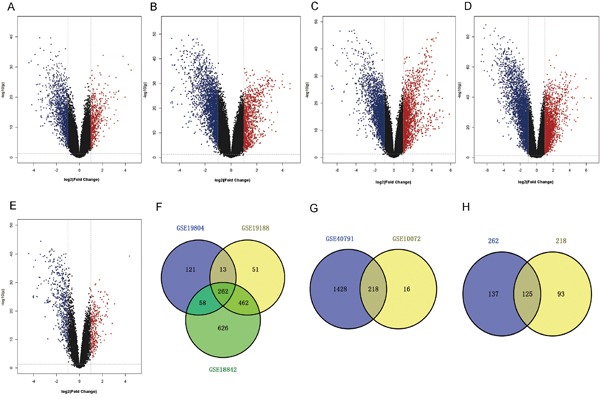
Identification of expression differences between tumor and NTL **A-E.** Volcano plot of the differential mRNA expression analysis. X-axis: log2 fold change; Y-axis: - log10 (FDR P-value) for each probes; Vertical dotted lines: fold change ≥ 2 or ≤ 2; Horizontal dotted line: the significance cutoff (FDR P-value = 0.05). (A) There were 1,388 genes identified to be differentially expressed in GSE19804, including 454 up-regulated and 934 down-regulated genes. (B) 2,421 genes (788 up-regulated and 1,633 down-regulated genes) differentially expressed in GSE19188. (C) 3,168 genes (1,403 up-regulated and 1,765 down-regulated genes) differentially expressed in GSE18842. (D) 3,796 genes (1,646 up-regulated and 2,150 down-regulated genes) differentially expressed in GSE40791. (E) 666 genes (234 up-regulated and 432 down-regulated genes) differentially expressed in GSE18842. **F-H.** Overlap analysis between different datasets. (F) A total of 262 genes were significantly differentially expressed in the three NSCLC datasets. (G) 218 genes were overlapped in the two LUAD datasets. (H) There were 125 overlapping genes significantly differentially expressed between tumor and NTL in all five datasets.

In our study, gene expression profiles from five datasets were utilized to compare gene expression between tumors and NTL. Three NSCLC gene expression profiles (GSE19804, GSE19188, GSE18842) and two LUAD gene expression profiles (GSE40791, GSE10072) were analyzed to identify the differentially expressed genes (DEGs) during tumorigenesis. Genes with corrected P-value < 0.05 and absolute fold change > 2 were considered as DEGs. The results showed that, 1,388 genes (454 up-regulated and 934 down-regulated genes) were identified to be differentially expressed in GSE19804, 2,421 genes (788 up-regulated and 1,633 down-regulated genes) differentially expressed in GSE19188, 3,168 genes (1,403 up-regulated and 1,765 down-regulated genes) in GSE18842, 3,796 genes (1,646 up-regulated and 2,150 down-regulated genes) in GSE40791 and 666 genes (234 up-regulated and 432 down-regulated genes) in GSE18842 (Figure 1A–1E). Then, we performed an exploratory two-dimensional hierarchical clustering of the differentially expressed probes, the mRNA expression profiles of tumors and NTL resulted in separate clusters (Supplementary Figure S1). Additionally, we conducted an overlapping analysis of the DEGs in NSCLC and LUAD to identify genes which were specifically overexpressed in LUAD. As indicated in Figure 1F, a total of 262 genes were significantly differentially expressed in the three NSCLC datasets. 218 genes were overlapped in the two LUAD datasets as shown in Figure 1G. After further screening by overlapping these two subsets of genes, 125 genes were identified that could be exclusive to LUAD carcinogenesis (Figure 1H, Supplementary Table S1).

### Function enrichment of differentially expressed genes

**Figure 2:**
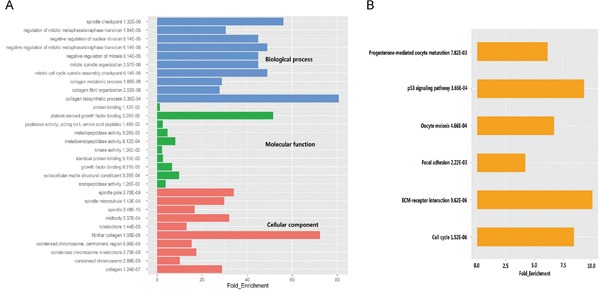
GO and pathway analysis of significant differentially expressed genes **A.** The top ten significantly enriched GO categories were calculated. Blue: Biological process; Green: Molecular function; Red: Cellular component. **B.** Gene networks identified through KEGG analysis of the differentially expressed genes.

To determine biological functions of the 125 DEGs, Gene Ontology (GO) analysis was performed. In terms of the three different domains of GO, genes associated with biological processes were mainly involved in cell cycle and mitosis, 5 of the top 10 enriched categories of molecular function were related to peptidase activity or kinase activity, while spindle and chromosome were the most enriched cellular components (Figure 2A, Supplementary Table S2). We further investigated the functional implication of these DEGs in the development of LUAD by Kyoto Encyclopedia of Genes and Genomes (KEGG) analysis. The results demonstrated that DEGs were enriched in six KEGG pathways. Cell cycle was the most significant one (P =1.52× 10^−6^), followed by ECM-receptor interaction (P = 9.62× 10^−6^), p53 signaling pathway (P = 3.66× 10^−4^) (Figure 2B, Supplementary Table S3). Most of enriched functional catalogues and pathways have been found to be closely associated with the incidence and development of cancer, which emphasized an implication of the DEGs in LUAD. We also found that several genes were repeatedly involved in cell cycle and mitosis, including MAD2L1 and CDK1.

### CDK1 and MAD2L1 were overexpressed in LUAD

**Figure 3:**
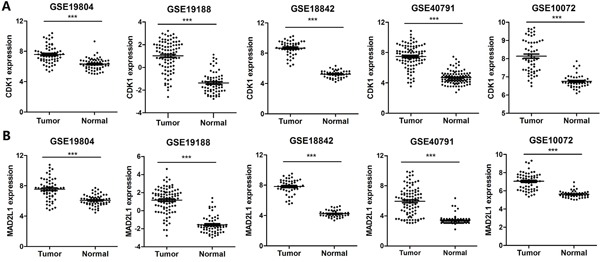
Identification of the differentially expressed genes **A.** Identification of mRNA expression of CDK1 in five datasets, respectively. **B.** Identification of mRNA expression of MAD2L1 in five datasets, respectively. *** corresponds to P <0.001; ** P <0.01 and * P <0.05.

**Figure 4:**
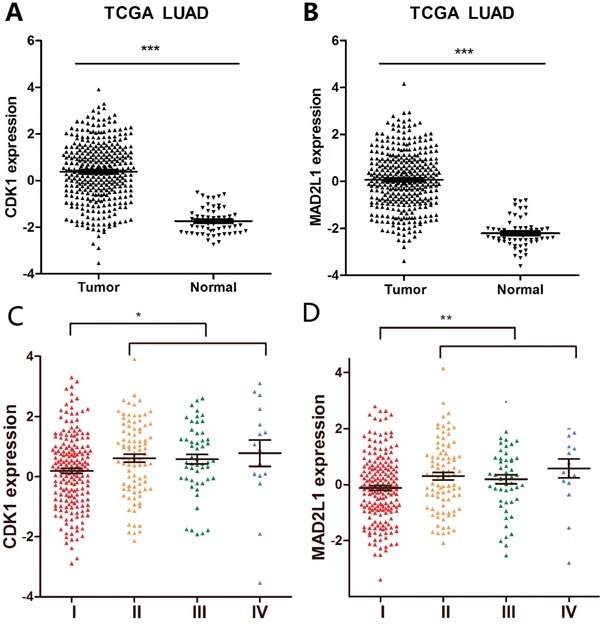
Validation of the differentially expressed genes **A.** Validation of mRNA expression of CDK1 in TCGA datasets. **B.** Validation of mRNA expression of MAD2L1 in TCGA datasets. **C.** Gene expression of CDK1 in LUAD patients according to clinical stage. **D.** Gene expression of MAD2L1 in LUAD patients according to clinical stage. *** corresponds to P <0.001; ** P <0.01 and * P <0.05.

CDK1 and MAD2L1 were selected for further study due to their known role in regulating tumor cell cycle and mitosis. The increased expression of CDK1 and MAD2L1 in LUAD was identified in five discovering datasets (three NSCLC datasets and two LUAD datasets) (Figure 3A and 3B). Furthermore, we validated their over-expression by using the Cancer Genome Atlas (TCGA) database. A total of 349 LUAD tissues and 58 NTL tissue samples were selected. The expression levels of the two selected genes were in line with those of our training cohorts, with significant differences between tumor and NTL (Figure 4A and 4B). All these results suggested that overexpression of CDK1 and MAD2L1 was a common feature for LUAD. We also found that CDK1 and MAD2L1 expression was elevated in patients with a higher pathological stage (Figure 4C and 4D).

### Associations of CDK1 and MAD2L1 expression with clinicopathological variables

Clinicopathological characteristics of the LUAD patients are listed in Table 1. CDK1 expression was remarkably positively associated with gender (P = 0.008), smoking history (P < 0.001), pathologic T stage (P = 0.001) and therapy outcome (P= 0.015). No significant difference of CDK1 mRNA levels was found in patients with different age, clinical stage and new tumor event (P >0.05). As Table 1 showed, the elevated MAD2L1 expression was significantly correlated with gender (P < 0.001), smoking history (P < 0.001), clinical stage (P = 0.016) and pathologic T stage (P = 0.001).

**Table 1:** Clinical characteristics and correlations with the expression of CDK1 and MAD2L1

Characteristic	n=349	CDK1			MAD2L1		
		Low (n=174)	High (n=175)	P value	Low (n=174)	High (n=175)	P value
**Age (years)**							
<65	159	74	85	0.257	71	88	0.075
≥65	190	100	90		103	87	
**Gender**							
Female	190	107	83	**0.008**	111	79	**<0.001**
Male	159	67	92		63	96	
**Smoking history**							
Current smoker	80	28	52	**<0.001**	27	53	**<0.001**
Current reformed smoker for ≤15 years	115	48	67		51	64	
Current reformed smoker for > 15 years	85	57	28		56	29	
Never-smoker	53	33	20		34	19	
**Clinical stage**							
I & II	281	146	135	0.111	149	132	**0.016**
III & IV	68	28	40		25	43	
**New tumor event**							
YES	99	40	59	0.26	41	58	**0.047**
NO	250	134	116		133	117	
**Pathologic T stage**							
T1	127	80	47	**0.001**	81	46	**0.001**
T2	180	77	103		77	103	
T3 + T4	40	16	24		16	24	
**Therapy outcome**							
CR+PR	145	83	62	**0.015**	77	68	0.098
SD+PD	86	35	51		36	50	

### Associations of CDK1 and MAD2L1 expression with overall and relapse-free survival

**Figure 5:**
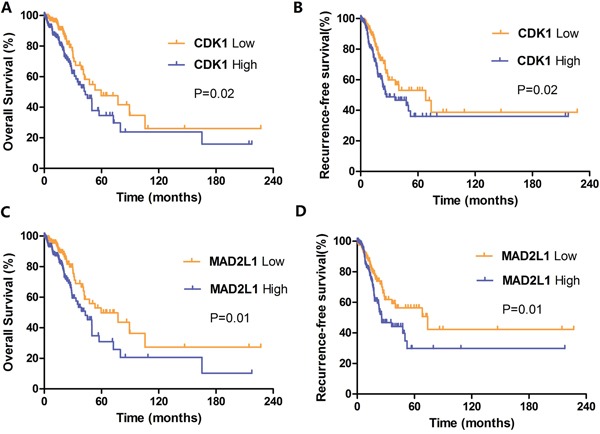
Kaplan-Meier survival curves by different levels of CDK1 and MAD2L1 expression in 349 LUAD patients **A.** Overall survival (OS) by low and high CDK1 expression; **B.** Relapse-free survival (RFS) by low and high CDK1 expression; **C.** Overall survival (OS) by low and high MAD2L1 expression; **D.** Relapse-free survival (RFS) by low and high MAD2L1 expression.

We next asked whether CDK1 and MAD2L1 expression will influence clinical outcomes of LUAD patients. As shown in Figure 5A and Figure 5B, CDK1 expression was significantly related with OS (P = 0.02) and RFS (P= 0.02) of LUAD patients. The median OS in CDK1 low expression group is 59.7 months, in CDK1 high expression group is 43.1 months. The median RFS in CDK1 low expression group is 68.2 months, in CDK1 high expression group is 26.9 months. Those figures meant that higher CDK1 expression indicated poorer prognosis and earlier recurrence. Similarly, elevated expression of MAD2L1 was both remarkably associated with reduced survival (P = 0.01; Figure 5C) and increased risk of recurrence (P = 0.01; Figure 5D). The median OS in low and high group of MAD2L1 is 59.7 months and 43.1 months. The median RFS in low and high group of MAD2L1 is 73.9 months and 25.7 months. In short, the overexpressed CDK1 and MAD2L1 have potentials to serve as prognostic biomarkers for prediction of LUAD recurrence and survival.

## DISCUSSION

High-throughput analyses are used to determine gene expression signatures for improved accuracy of prognosis [10]. In order to identify potential biomarkers for LUAD prognosis and therapy, we integrated expression profiles of 668 lung cancer patients in five datasets from the Gene Expression Omnibus (GEO) and identified a panel of differentially expressed genes (DEGs). Function enrichment analysis highlighted that these genes were closely related to the carcinogenesis of LUAD, such as cell cycle, ECM-receptor interaction and p53 signaling pathway. CDK1 and MAD2L1, two critical mitotic checkpoint genes, were selected for further study. Elevated expression of CDK1 and MAD2L1 was validated via an independent LUAD cohort. Kaplan-Meier analysis revealed that CDK1 and MAD2L1 correlated with both overall survival (OS) and relapse-free survival (RFS).

The cell cycle is an evolutionarily conserved process necessary for mammalian cell growth and development. Loss of normal cell-cycle control is a hallmark of human cancer [11]. Recently, many therapeutic strategies have been proposed for targeting the cell cycle in cancer. Chromosomal instability correlates with poor prognosis in multiple solid tumors, indicating that increasing genetic diversity contributes to changed tumor survival and chemoresistance [12]. At present, several cell cycle related genes have been reported to be involved in lung cancer initiation and development. Ding et al. [13] discovered that CCNB1 was a biomarker for the prognosis of ER+ breast cancer and monitoring of hormone therapy efficacy. Qian et al. [14] found that overexpression of CCNB2 protein was associated with clinical progression and poor prognosis in NSCLC. Another research showed that ISL1 promoted tumor cell proliferation and tumorigenesis. ISL1 serves as a novel regulator for the expression of CCNB1, CCNB2 and C-MYC, which plays significant roles in gastric cancer progression and development [15].

Cyclin-dependent kinases (CDKs) are important cell cycle-regulating proteins [16]. CDK1 is essential for cell cycle progression and proliferation, and the dysregulation of CDK1 activity was a common event in a variety of tumors [17–19]. The results in this study were similar to those published by others to date, which showed higher CDK1 expression and activity in prostate cancer, colorectal cancer and epithelial ovarian cancer [20–22]. Another potential prognostic factor for LUAD is MAD2L1. MAD2L1 is required in mitosis when chromosomes are unattached to the mitotic spindle that maintains chromosomal segregation, and is involved in the spindle checkpoint during mitosis [23–25]. Dysregulation of MAD2L1 is associated with chromosomal instability and substantial aneuploidy which frequently occur in cancer [26].

In conclusion, the present study has shown that CDK1 and MAD2L1 are overexpressed in LUAD tissues and that its up-regulation may be indicative of poor survival rates and a higher risk for cancer recurrence, and it could have a potential role as a prognostic marker in LUAD patients. Functional researches are necessary to reveal the molecular mechanisms of CDK1 and MAD2L1 in LUAD and their role in prognosis and therapeutic target.

## MATERIALS AND METHODS

All data analysis were performed using R (http://www.r-project.org/, version 2.15.0) and Bioconductor [27].

### Lung cancer patient datasets

Training datasets (GSE19804, GSE19188, GSE18842, GSE40791, GSE10072) based on the Affymetrix platform (Affymetrix HG-U133 Plus 2.0 array and HG-U133A array) and corresponding clinical information of lung cancer patients were retrieved from Gene Expression Omnibus (http://www.ncbi.nlm.nih.gov/geo). Three NSCLC genome-wide expression profiles were extracted from the following three datasets: GSE19804, which includes 60 paired tumor and normal tissues, GSE19188, including 91 tumor and 65 adjacent normal lung tissues, and GSE18842, which includes 46 tumors and 45 controls. Two LUAD genome-wide expression profiles were extracted from GSE40791 and GSE10072, with the former including 94 tumor and 100 non-tumor tissues and the latter containing 58 tumor and 49 non-tumor tissues.

Validation datasets were downloaded from the Cancer Genome Atlas (TCGA) data portal (http://tcga-data.nci.nih.gov). We selected 349 tumor and 58 NTL samples, with both mRNA expression data and clinical features information available for performing the correlation analysis and survival analysis. Detailed clinical information of patients used in this study was shown in Table 1.

### Global gene expression analysis

Raw microarray data files (.CEL files) of the five patient datasets were downloaded from the GEO database. Background correction and quartile normalization were performed using the Robust Multichip Average (RMA) algorithm by the R package Affy [28, 29]. After that, the Linear Models for Microarray Data (LIMMA) package in R was used to calculate the probability of probes being differentially expressed between cases and controls. The fold change (FC) and its logarithm value (log FC) were also determined. Corrected P-value < 0.05 and absolute fold change > 2 were used to identify significantly differential expressed mRNAs. The heat map, locus-by-locus volcano plot and venn diagram of significant differentially expressed mRNA were performed by gplots, lattice, and venn diagram packages in R, respectively [30].

### Functional enrichment analysis

The Database for Annotation, Visualization and Integrated Discovery (DAVID) bioinformatics resource consists of an integrated biological knowledge base and analytic tools aimed at systematically extracting biological meaning from large gene or protein lists [31, 32]. In the present study, DAVID was applied to conduct Kyoto encyclopedia of genes and genomes (KEGG) pathway and gene ontology (GO) enrichment analyses for the identified target genes. KEGG is a knowledge base for systematic analysis of gene functions. GO analysis predicts the function of the target genes in three aspects, including biological processes, cellular components and molecular function. Functional annotation with a P-value < 0.05 and an enrichment score > 2.0 were considered statistically significant.

### Statistical analysis

Statistical analyses were performed with SPSS version 18.0 and GraphPad Prism 5.0 software. Single comparisons between two groups were determined by Student's t-test. Survival analysis was carried out according to Kaplan–Meier analysis and Log-rank test. Two types of survival outcomes were included in survival analysis: overall survival (OS), defined as the time between the date of surgery and date of death or last follow-up, and relapse-free survival (RFS), defined as the period from surgery to recurrence or last follow-up. P-values less than 0.05 were considered statistically significant.

## SUPPLEMENTARY MATERIALS FIGURE AND TABLES




