# Supplementary material for: Prognostic and predictive values of CDK1 and MAD2L1 in lung adenocarcinoma
Source: Oncotarget. 2016 Nov 9;7(51):85235–43. doi: 10.18632/oncotarget.13252 (PMC5356732; doi:10.18632/oncotarget.13252)
Supplement: Supplementary file 1 [file oncotarget-07-85235-s001.pdf]

Prognostic and predictive values of CDK1 and MAD2L1 in lung adenocarcinoma

SUPPLEMENTARY FIGURE AND TABLES

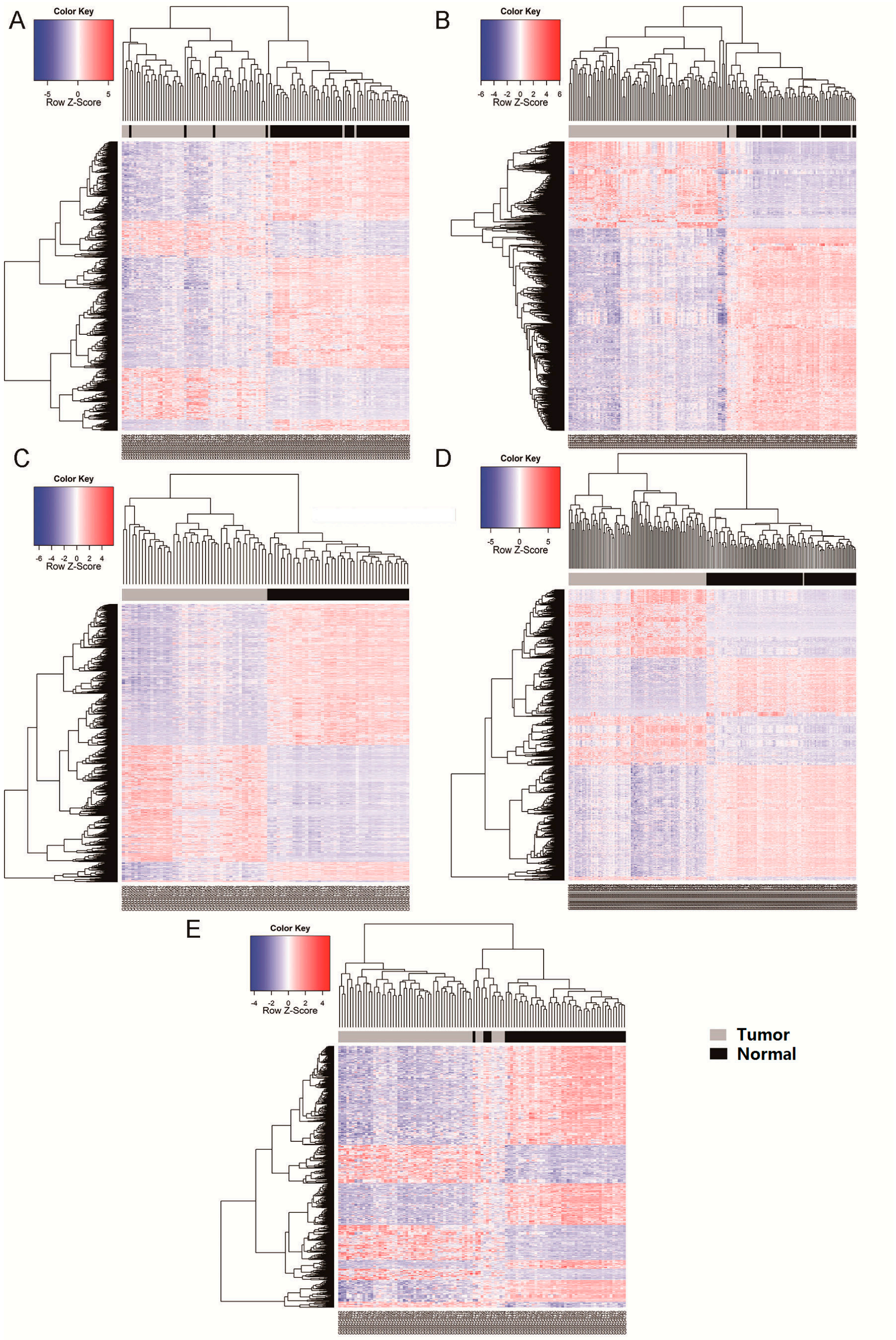

Supplementary Figure S1: Two-dimensional hierarchical clustering of the significant and differentially expressed probes in all samples. Probes are in rows; samples are in columns. Both up-regulated and down-regulated mRNAs can be seen in tumors compared with normal tissues. **A.** GSE19804; **B.** GSE19188; **C.** GSE18842; **D.** GSE40791; **E.** GSE10072.

Supplementary Table S1: Differently expressed genes (DEGs) in in LUAD

See Supplementary File 1

Supplementary Table S2: Top ten significantly enriched GO categories in LUAD

GO Terms	*P* value	Fold Enrichment
**Biological Processes**		
collagen biosynthetic process	5.30E-04	80.66
spindle checkpoint	1.32E-06	56.01
negative regulation of mitotic metaphase/anaphase transition	6.14E-05	48.88
mitotic cell cycle spindle assembly checkpoint	6.14E-05	48.88
negative regulation of mitosis	8.14E-05	44.81
negative regulation of nuclear division	8.14E-05	44.81
mitotic spindle organization	3.57E-06	44.81
regulation of mitotic metaphase/ anaphase transition	1.84E-05	30.55
collagen metabolic process	1.69E-06	28.8
collagen fibril organization	2.03E-06	27.81
**Molecular Function**		
platelet-derived growth factor binding	5.28E-05	51.46
extracellular matrix structural constituent	3.39E-04	9.87
metalloendopeptidase activity	8.12E-04	8.16
protein binding	0.001	1.28
endopeptidase activity	0.001	3.77
identical protein binding	0.005	2.65
growth factor binding	0.006	6.73
metallopeptidase activity	0.009	4.64
kinase activity	0.013	2.2
peptidase activity, acting on L-amino acid peptides	0.014	2.57
**Cellular Component**		
fibrillar collagen	1.05E-08	72.3
spindle pole	2.70E-09	34.02
midbody	2.37E-04	32.13
spindle microtubule	1.43E-06	29.92
collagen	1.24E-07	28.92
condensed chromosome kinetochore	2.73E-06	17.45
spindle	3.48E-15	16.72
condensed chromosome, centromeric region	5.86E-06	15.33
kinetochore	1.44E-05	13.14
condensed chromosome	2.88E-06	10.08

Supplementary Table S3: The top six significantly enriched pathways in LUAD

Terms	P Value	Fold Enrichment	Genes
hsa04110:Cell cycle	1.52E-06	8.47	CCNB1, CDK1, MAD2L1, CCNB2, CDKN2A, BUB1, BUB1B, TTK, CDC20, MCM4
hsa04512:ECM-receptor interaction	9.62E-06	10.08	COL3A1, COL1A2, COL1A1, COL5A2, THBS2, COL11A1, COL5A1, SPP1
hsa04115:p53 signaling pathway	3.66E-04	9.34	CCNB1, CDK1, CCNB2, CDKN2A, RRM2, IGFBP3
hsa04114:Oocyte meiosis	4.66E-04	6.74	CCNB1, CDK1, MAD2L1, CCNB2, BUB1, CDC20, AURKA
hsa04510:Focal adhesion	0.002	4.21	COL3A1, COL1A2, COL1A1, COL5A2, THBS2, COL11A1, COL5A1, SPP1
hsa04914:Progesterone-mediated oocyte maturation	0.007	6.15	CCNB1, CDK1, MAD2L1, CCNB2, BUB1
