# Supplementary material for: Prognostic and predictive values of CDK1 and MAD2L1 in lung adenocarcinoma
Source: Oncotarget. 2016 Nov 9;7(51):85235–43. doi: 10.18632/oncotarget.13252 (PMC5356732; doi:10.18632/oncotarget.13252)
Supplement: Supplementary file 2 [file oncotarget-07-85235-s002.doc]

Supplementary Table 1 Differently expressed genes (DEGs) in in LUAD.

| Gene symbol | Gene name |
| --- | --- |
| ADAM28 | ADAM metallopeptidase domain 28 |
| AK4 | Adenylate kinase 4 |
| ANP32E | Acidic (leucine-rich) nuclear phosphoprotein 32 family, member E |
| ASPM | Asp (abnormal spindle) homolog, microcephaly associated (Drosophila) |
| AURKA | Aurora kinase A |
| BIRC5 | Baculoviral IAP repeat containing 5 |
| BUB1 | BUB1 mitotic checkpoint serine/threonine kinase |
| BUB1B | BUB1 mitotic checkpoint serine/threonine kinase B |
| C1orf106 | Chromosome 1 open reading frame 106 |
| CCNB1 | Cyclin B1 |
| CCNB2 | Cyclin B2 |
| CD24 | CD24 molecule |
| CDC20 | Cell division cycle 20 |
| CDH3 | Cadherin 3, type 1, P-cadherin (placental) |
| CDK1 | Cyclin-dependent kinase 1 |
| CDKN2A | Cyclin-dependent kinase inhibitor 2A |
| CDKN3 | Cyclin-dependent kinase inhibitor 3 |
| CEACAM5 | Carcinoembryonic antigen-related cell adhesion molecule 5 |
| CENPA | Centromere protein A |
| CENPF | Centromere protein F, 350/400kDa |
| CENPU | Centromere protein U |
| CEP55 | Centrosomal protein 55kDa |
| COL10A1 | Collagen, type X, alpha 1 |
| COL11A1 | Collagen, type XI, alpha 1 |
| COL1A1 | Collagen, type I, alpha 1 |
| COL1A2 | Collagen, type I, alpha 2 |
| COL3A1 | Collagen, type III, alpha 1 |
| COL5A1 | Collagen, type V, alpha 1 |
| COL5A2 | Collagen, type V, alpha 2 |
| CP | Ceruloplasmin (ferroxidase) |
| CRABP2 | Cellular retinoic acid binding protein 2 |
| CST1 | Cystatin SN |
| CXCL13 | Chemokine (C-X-C motif) ligand 13 |
| CXCL14 | Chemokine (C-X-C motif) ligand 14 |
| DLGAP5 | Discs, large (Drosophila) homolog-associated protein 5 |
| DNAJC12 | DnaJ (Hsp40) homolog, subfamily C, member 12 |
| DSP | Desmoplakin |
| ECT2 | Epithelial cell transforming 2 |
| EFNA4 | Ephrin-A4 |
| ENO1 | Enolase 1, (alpha) |
| EZH2 | Enhancer of zeste 2 polycomb repressive complex 2 subunit |
| FAP | Fibroblast activation protein, alpha |
| FEN1 | Flap structure-specific endonuclease 1 |
| FHL2 | Four and a half LIM domains 2 |
| FKBP11 | FK506 binding protein 11, 19 kDa |
| GALNT7 | Polypeptide N-acetylgalactosaminyltransferase 7 |
| GCNT3 | Glucosaminyl (N-acetyl) transferase 3, mucin type |
| GGCT | Gamma-glutamylcyclotransferase |
| GINS1 | GINS complex subunit 1 (Psf1 homolog) |
| GMNN | Geminin, DNA replication inhibitor |
| GOLM1 | Golgi membrane protein 1 |
| GPR87 | G protein-coupled receptor 87 |
| GREM1 | Gremlin 1, DAN family BMP antagonist |
| HIST1H2BD | Histone cluster 1, H2bd |
| HMGB3 | High mobility group box 3 |
| HN1 | Hematological and neurological expressed 1 |
| IGF2BP3 | Insulin-like growth factor 2 mRNA binding protein 3 |
| IGFBP3 | Insulin-like growth factor binding protein 3 |
| IGHM | Immunoglobulin heavy constant mu |
| IGHV3-23 | Immunoglobulin heavy variable 3-23 |
| IGK | Immunoglobulin kappa locus |
| IGKV4-1 | Immunoglobulin kappa variable 4-1 |
| IGL | Immunoglobulin lambda locus |
| IGLJ3 | Immunoglobulin lambda joining 3 |
| IGLV1-40 | Immunoglobulin lambda variable 1-40 |
| KDELR2 | KDEL (Lys-Asp-Glu-Leu) endoplasmic reticulum protein retention receptor 2 |
| KIAA0101 | KIAA0101 |
| KIF11 | Kinesin family member 11 |
| KIF4A | Kinesin family member 4A |
| KRT15 | Keratin 15 |
| LAPTM4B | Lysosomal protein transmembrane 4 beta |
| LGR4 | Leucine-rich repeat containing G protein-coupled receptor 4 |
| LGSN | Lengsin, lens protein with glutamine synthetase domain |
| LOC100133862///IGHM | Ig heavy chain V-I region V35-like///immunoglobulin heavy constant mu |
| LOC100510044///IGK | Immunoglobulin kappa locus-like///immunoglobulin kappa locus |
| MAD2L1 | MAD2 mitotic arrest deficient-like 1 |
| MCM4 | Minichromosome maintenance complex component 4 |
| MDK | Midkine (neurite growth-promoting factor 2) |
| MELK | Maternal embryonic leucine zipper kinase |
| MMP1 | Matrix metallopeptidase 1 (interstitial collagenase) |
| MMP11 | Matrix metallopeptidase 11 (stromelysin 3) |
| MMP12 | Matrix metallopeptidase 12 (macrophage elastase) |
| MMP9 | Matrix metallopeptidase 9 (gelatinase B, 92kDa gelatinase, 92kDa type IV collagenase) |
| NDC80 | NDC80 kinetochore complex component |
| NEK2 | NIMA-related kinase 2 |
| NME1 | NME/NM23 nucleoside diphosphate kinase 1 |
| NMU | Neuromedin U |
| NQO1 | NAD(P)H dehydrogenase, quinone 1 |
| NUSAP1 | Nucleolar and spindle associated protein 1 |
| PAICS | Phosphoribosylaminoimidazole carboxylase, phosphoribosylaminoimidazole succinocarboxamide synthetase |
| PBK | PDZ binding kinase |
| PCP4 | Purkinje cell protein 4 |
| PLAU | Plasminogen activator, urokinase |
| PLOD2 | Procollagen-lysine, 2-oxoglutarate 5-dioxygenase 2 |
| POU2AF1 | POU class 2 associating factor 1 |
| PPAP2C | Phosphatidic acid phosphatase type 2C |
| PRC1 | Protein regulator of cytokinesis 1 |
| RCC1 | Regulator of chromosome condensation 1 |
| RGS17 | Regulator of G-protein signaling 17 |
| RRM2 | Ribonucleotide reductase M2 |
| S100A2 | S100 calcium binding protein A2 |
| SCG5 | Secretogranin V (7B2 protein) |
| SLC2A1 | Solute carrier family 2 (facilitated glucose transporter), member 1 |
| SLC7A11 | Solute carrier family 7 (anionic amino acid transporter light chain, xc- system), member 11 |
| SORD | Sorbitol dehydrogenase |
| SOX4 | SRY (sex determining region Y)-box 4 |
| SPAG4 | Sperm associated antigen 4 |
| SPINK1 | Serine peptidase inhibitor, Kazal type 1 |
| SPP1 | Secreted phosphoprotein 1 |
| SRD5A1 | Steroid-5-alpha-reductase, alpha polypeptide 1 (3-oxo-5 alpha-steroid delta 4-dehydrogenase alpha 1) |
| SRPX2 | Sushi-repeat containing protein, X-linked 2 |
| SULF1 | Sulfatase 1 |
| TCN1 | Transcobalamin I (vitamin B12 binding protein, R binder family) |
| TFAP2A | Transcription factor AP-2 alpha (activating enhancer binding protein 2 alpha) |
| THBS2 | Thrombospondin 2 |
| TMPRSS11E | Transmembrane protease, serine 11E |
| TMPRSS4 | Transmembrane protease, serine 4 |
| TOP2A | Topoisomerase (DNA) II alpha 170kDa |
| TOX3 | TOX high mobility group box family member 3 |
| TPBG | Trophoblast glycoprotein |
| TPX2 | TPX2, microtubule-associated |
| TTK | TTK protein kinase |
| TYMS | Thymidylate synthetase |
| UBE2C | Ubiquitin-conjugating enzyme E2C |
| ZWINT | ZW10 interacting kinetochore protein |
